# The antioxidant activity and nitric oxide production of extracts obtained from the leaves of Chenopodium quinoa Willd

**DOI:** 10.1051/bmdcn/2017070424

**Published:** 2017-11-24

**Authors:** Hsiao-Ling Chen, Xiang-Zhen Lan, Yan-Yi Wu, Yu-Wen Ou, Tsung Chi Chen, Wen-Tzu Wu

**Affiliations:** 1 Department of Food Nutrition and Health Biotechnology, Asia University Taichung 413 Taiwan; 2 Department of Biotechnology, Asia University Taichung 413 Taiwan

**Keywords:** Chenopodium quinoa, Antioxidant activity, Nitric oxide

## Abstract

Background: Most reports have indicated the antioxidant capacity of quinoa seeds. However, the leaves of Quinoa (*Chenopodium quinoa* Willd.) are usually worthless and little known about their biological activities. In this study, the antioxidant and immunomodulatory potential of the quinoa leaf extracts were explored.

Methods: The crude leaf extracts of quinoa were extracted using water, 50% ethanol or 95% ethanol as solvent, denoted WQL, 50% EQL and 95% EQL, respectively. The antioxidant activities of quinoa leaf extracts were assessed by the ability of 1,1-diphenyl-2-picrylhydrazyl (DPPH) scavenging and iron chelating. The total phenolic content was determined. Inhibition of nitric oxide (NO) production in the lipopolysaccharide (LPS)-induced murine macrophage RAW 264.7 cells was examined to gauge the anti-inflammatory activity.

Results: The 95% EQL showed a higher level of total phenolic content (569.5 mg GAE/g extract) and better DPPH scavenging activity. The WQL exhibited a better iron chelating capacity (28.9% at 10 mg/*ml*). The iron chelating activity of the 95% EQL increased in a concentration-dependent manner, which ranged from 10.9% up to 53.9%. The 50% EQL and 95% EQL significantly inhibited NO production in the LPSstimulated RAW 264.7 cells.

Conclusion: We demonstrate that the extracts of quinoa leaves possess the biological activities of antioxidant and anti-inflammatory. Our finding suggests that the leaf extract of quinoa has potential to be utilized for natural health products.

## 1. Introduction

A large amount of scientific evidence suggests that oxidative stress is involved in the pathogenesis of numerous human diseases. The natural antioxidants from plant extracts are the subject of increasing scientific interest because of their function in the prevention of disease. Most of the research on the potential effects has been focused on free radical scavenging, metal chelating, anti-inflammation, and anticancer activities [1, 2]. All parts of plants, including their leaves, bark, roots, flowers, fruits, and seeds have been extensively studied for their antioxidant activity. The phenolic compounds are widely available in plants and have been reported as the main contributors to the antioxidative and diverse biological properties [3].

The genus Chenopodium includes more than 200 species and many of these are edible either whole plants or parts of the plant. The widely used of Chenopodium species in traditional medicine have resulted in its phytochemicals, such as flavonoids [4]. Quinoa *(Chenopodium quinoa* Willd.) is known as a pseudo-cereal and has been found to contain compounds like polyphenols, phytosterols, and flavonoids, all with possible nutraceutical benefits. It is traditionally consumed by the native population of the Andes region [5] and has been recognized as a complete food that is rich in nutrients [6]. With the increasing worldwide attention to its nutritional contents, the quinoa seeds have been reported to exhibit beneficial effects on human health. However, very few studies exist conducted on the biological activities of quinoa leaves. Quinoa leaves till now have been thought of as worthless waste but are actually edible and may serve as a valuable ingredient in functional food. The aim of this study was to evaluate the potential bioactivity of quinoa leaf extracts on antioxidant and anti-inflammation activities. The antioxidant activities of these extracts were determined by using DPPH scavenging and the chelation capacity of ferrous ion. The production of nitric oxide (NO) induced by lipopolysaccharide (LPS) in RAW 264.7 cells was used to assess the anti-inflammatory effects.

## 2. Materials and methods

### 2.1. Chemicals and plant materials

1,1-Diphenyl-2-picrylhydrazyl (DPPH), Folin-Ciocalteu’s reagent, gallic acid, lipopolysaccharide (LPS), ferrozine, sodium carbonate, and dimethyl sulfoxide (DMSO) were purchased from Sigma-Aldrich (St. Louis, MO, USA). RPMI 1640, fetal bovine serum (FBS), antibiotic antimycotic solution, phosphate buffered saline (PBS), and 3-[4, 5-dimethylthiazole-2-yl]-2,5-diphenyltetrazolium bromide (MTT) were purchased from Gibco (Thermo-Fisher Science, Inc.). Ethanol (analytical grade) was purchased from ECHO Chemical Co., Ltd. (Miaoli, Taiwan). Quinoa plants were grown in a temperature-controlled greenhouse. Fully expanded leaves of quinoa were harvested and freeze-dried for 24-h. The dried sample was ground into powder using a kitchen milling machine and stored at -20°C prior to extraction.

### 2.2. Preparation of crude extracts

The quinoa leaf powder (1 g) was extracted with 50 *ml* of distilled water, 50% and 95% ethanol for 16-h at room temperature, and the solid-liquid mixture was filtered through Whatman No.2 filter paper (ADVANTEC qualitative filter paper number 2). The resulting supernatant was concentrated under a vacuum at 40°C (EYELA rotary evaporator N-1000, Japan) and freeze-dried for 24-h to obtain the crude extracts. The dried water extract was dissolved in PBS, denoted WQL. Dried extracts of quinoa leaves using 50% and 95% ethanol were dissolved in DMSO and named 50% EQL and 95% EQL, respectively. The stock solutions of 100 mg/ml were stored at -20°C prior to determining the antioxidation activity and the inhibition of NO production.

### 2.3. Determination of total phenolic content

The total phenolic contents of WQL, 50% EQL and 95% EQL were determined by the Folin-Ciocalteau assay [7]. Each diluted extract sample or gallic acid (0.5 *ml)* was mixed with 2.5 *ml* of Folin’s reagent (diluted with distilled water 1:10) and 2 *ml* of 7.5% (w/v) sodium bicarbonate solution. Their absorbance was determined spectrophotometrically at 760 nm using a microplate reader (Epoch 2, BioTek, VT, USA) after standing for 30 min at room temperature. The results were expressed as milligram gallic acid equivalent (GAE) per gram extract.

### 2.4. Free radical scavenging assay

The DPPH radical-scavenging activity of all crude extracts of quinoa leaves was determined according to the method of Shimada *et al.* [8] with slight modifications. The crude extract (50 μL) with varying concentrations (10, 50 and 100 mg/ml) was added to 100 μL of 1 mM DPPH in methanol. This mixture was shaken and allowed to stand for 30 min at room temperature in the dark. The absorbance was measured at 517 nm using a microplate reader (Epoch 2, BioTek). The percentage inhibition of radicals was calculated as [(*A*_control_ - *A*_sample_) / *A*_control_] × 100%, where *A*_control_ is the absorbance of DPPH solution without extract and *A*_sample_ is the absorbance of the sample with DPPH solution.

### 2.5. Iron chelating assay

The chelation of ferrous ions by quinoa leaf extracts was estimated using the method reported by Dinis *et al.* [9]. Briefly, 25 μL of 2 mM FeCl_2_ was added to 0.25 *ml* of different concentrations of the crude extract (10, 50 and 100 mg/ml) and 0.05 *ml* of 5 mM ferrozine solution. The mixture was vigorously shaken and allowed to stand at room temperature for 10 min. The absorbance of the solution was thereafter measured at 562 nm and the inhibition percentage of ferrozine–Fe^2+^ complex formation was calculated as the same as the DPPH method described above.

### 2.6. Cell culture

The murine macrophage RAW 264.7 cell line was a gift from Dr. Yuan-Yen Chang. The cells were cultured in RPMI 1640 supplemented with 10% FBS and 1% antibiotic antimycotic solution at 37°C in a humidified incubator under 5% CO_2_.

### 2.7. Cell viability assay

Cell viability was determined by the MTT assay. Briefly, cells were seeded in a 96-well plate at a density of 5 × 10^4^ cells/well and allowed to adhere for 24-h before treatment. Next, cells were treated with various concentrations of WQL, 50% EQL, and 95% EQL, respectively. After 24-h incubation, MTT was added to a final concentration of 0.5 mg*/ml* and the cells were incubated for 2-h at 37°C. The medium was then removed and 150 *ml* of DMSO was added to dissolve the formazan precipitate. The absorbance was measured at 570 nm using a microplate reader (Epoch 2, BioTek). The cell viability was normalized to the medium control group and expressed as % of control. The non-cytotoxic dose of all crude extracts was determined to be 1-100 μg/*ml*, and 1, 10 and 100 μg/*ml* of all crude extracts were used for exposure in the subsequent experiments.

### 2.8. Inhibition of NO production

Cells in 96-well plates (5 × 10^4^ cells/well) were treated with WQL, 50% EQL, and 95% EQL at a final concentration as described in the previous section. After a 1-h treatment, cells were stimulated with 1 μg/*ml* of LPS for 24-h. Nitrite content in cell culture media was determined by the Griess reaction. Briefly, 100 μL of the conditioned media with an equal volume of Griess reagent (1% sulfanilamide, 0.1% NED and 2.5% H_3_PO_4_) in a 96-well plate was incubated at room temperature for 10 min. The absorbance at 540 nm was determined by a microplate reader (Epoch 2, BioTek). The production of nitrite was calculated from a sodium nitrite (NaNO_2_) standard curve.

### 2.9. Statistical analysis

All experiments were performed in triplicate and results were expressed as means ± SEM. The statistical analyses were carried out with SPSS version 12.0 (SPSS, Inc., Chicago, IL) using oneway ANOVA followed by Duncan’s test. Values were considered significantly different when *p* < 0.05.

## 3. Results

### 3.1. Total phenolic content

The total phenolic content of the crude extract of quinoa leaves is shown in Table 1 and is expressed as milligrams of gallic acid equivalents (GAE) per gram of dry extract. The highest amount of phenolic content was found in the 95% EQL (569.5 ± 69.5 mg GAE/g).

**Table 1:** Total phenolic content of crude extracts of the quinoa leaves.

Sample	Total phenolic content (mg GAE/g extract)
WQL	340.4 ± 26.6^a^
50% EQL	413.6 ± 72.6^ab^
95% EQL	569.5 ± 69.5^b^

WQL = water extract of quinoa leaves; 50% EQL = 50% ethanolic extract of quinoa leaves; 95% EQL = 95% ethanolic extract of quinoa leaves;

GAE = gallic acid equivalent. Mean values followed by different superscripts are significantly different (p < 0.05).

### 3.2. DPPH radical scavenging activity

**Fig. 1:**
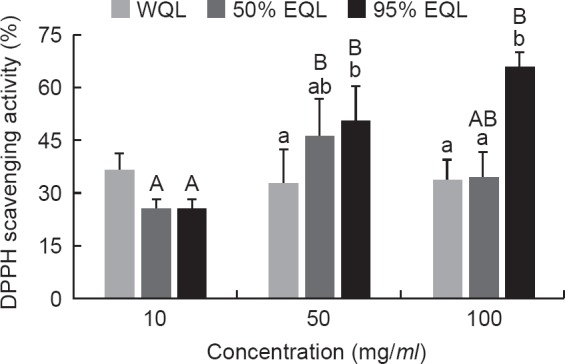
- The DPPH scavenging activity of the crude extracts from quinoa leaves. The data were mean ± SEM from three independent experiments. Different letters showed statistically significant differences (*p* < 0.05) among three extracts (lower case letters) and among three concentrations (capital letters) as analyzed by one-way ANOVA followed by Duncan’s test. WQL = water extract of quinoa leaves; 50% EQL = 50% ethanolic extract of quinoa leaves; 95% EQL = 95% ethanolic extract of quinoa leaves.

The radical scavenging ability of the crude extracts of quinoa leaves was measured using the DPPH scavenging assay. The results showed that the 50% EQL and 95% EQL were able to scavenge DPPH radicals in a concentration-dependent manner (Fig. 1). In the concentration of 50 and 100 mg/*ml*, the 95% EQL exhibited the highest level of DPPH radical scavenging activity, which were 50.7 ± 9.7% and 65.3 ± 4.7%, respectively.

### 3.3. Iron chelating activity

**Fig. 2:**
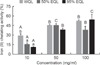
- The iron chelating activity of the crude extracts from quinoa leaves. The data were mean ± SEM from three independent experiments. Different letters showed statistically significant differences *(p* < 0.05) among three extracts (lower case letters) and among three concentrations (capital letters) as analyzed by one-way ANOVA followed by Duncan’s test. WQL = water extract of quinoa leaves; 50% EQL = 50% ethanolic extract of quinoa leaves; 95% EQL = 95% ethanolic extract of quinoa leaves.

In the concentration of 10 mg*/ml,* the WQL showed a significantly higher iron chelating ability (28.9 ± 2.8%) than the 50% EQL and 95% EQL (Fig. 2). The level of iron chelating ability in the 95% EQL was observed in a concentration-dependent manner. The WQL and 50% EQL showed the highest iron chelating activity at 50 mg*/ml* than their counterparts at 10 mg*/ml* and 100 mg/*ml*.

### 3.4. Cell viability

**Fig. 3:**
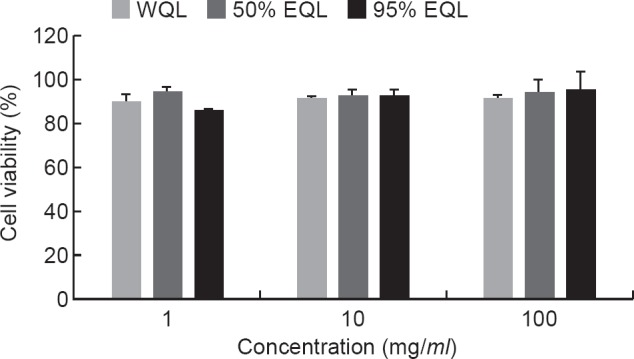
- The cell viability of the crude extracts in the RAW 264.7 cells. The RAW 264.7 cells were cultured in the presence of WQL, 50% EQL, and 95% EQL at indicated concentrations (1, 10 and 100 *yuglmt)* for 1-h, and then 1 pglml LPS for 24-h. The cell viability was measured *via* MTT assay. Values are means ± SEM (n = 3). WQL = water extract of quinoa leaves; 50% EQL = 50% ethanolic extract of quinoa leaves; 95% EQL = 95% ethanolic extract of quinoa leaves.

The effects of pretreated with different concentrations (1, 10, 100 μg/*ml*) of WQL, 50% EQL, and 95% EQL for 1-h and then exposed simultaneously to 1 μg/*ml* LPS for 24-h, the cell viability was determined by MTT assay. The cell viability was not influenced by the quinoa leaf extracts and LPS treatment (Fig. 3). Our result showed that no significant cytotoxicity on the RAW 264.7 cells was observed even up to 100 μg/*ml* of all crude extracts was treated.

### 3.5. Inhibition of NO production in RAW 264.7 cells

**Fig. 4:**
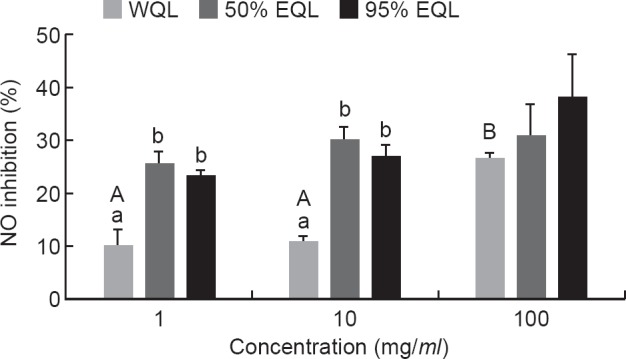
- The effect of the crude extracts on the NO production of the RAW 264.7 cells. After pre-treatment of the cells with different concentrations of the crude extracts (1,10 and 100 pglml) for 1-h, then the 1 pglml LPS was added to the cells for 24-h. Values are means ± SEM (n = 3). Different letters showed statistically significant differences (p < 0.05) among three extracts (lower case letters) and among three concentrations (capital letters) as analyzed by one-way ANOVA followed by Duncan’s test. WQL = water extract of quinoa leaves; 50% EQL = 50% ethanolic extract of quinoa leaves; 95% EQL = 95% ethanolic extract of quinoa leaves.

Pretreatment with the WQL, 50% EQL and 95% EQL at indicated concentrations (1, 10 and 100 μg/*ml*) for 1-h, the inhibition of the LPS-induced NO production ranged from 10.2% to 38.1% (Fig. 4). The WQL showed significantly higher NO inhibition at the concentration of 100 μg/*ml* as compared to its counterparts at 1 and 10 μg/*ml*, respectively. In a comparison of the three different extracts at 1 and 10 μg/*ml*, the 50% EQL and 95% EQL showed significantly higher inhibition of NO production in LPS-stimulated RAW 264.7 cells than WQL.

## 4. Discussion

The antioxidant potential of plant extracts has been widely tested by using various assays *in vitro.* The radical-scavenging assay and chelate transition metals have gained acceptance among researchers for their capacity to rapidly screen materials of interest. Iron is well known to be a powerful initiator for free radical oxidation and serves as a reagent for the Fenton reaction and lipid peroxidation that are the cause of many diseases, such as cancer [10]. In this study, the DPPH scavenging activity showed a correlation with the total phenolic content in the crude extracts, something that has been similarly reported by previous researches [1, 11]. Similar results have been reported by Ajayi *et al.* in aqueous leaf extracts of Chenopodium opulifolium leaves [12]. These indicates that phenolic compounds in the leaf extracts may be responsible for the antioxidant potential of Chenopodium species. Phenolics are powerful antioxidants and can delay or inhibit oxidative processes by chelating transition metals that play vital roles in the initiation of deleterious free radical reactions [13].

The results of the present study demonstrated that the crude leaf extracts of quinoa exhibited chelating activity by reducing the formation of ferrozine-Fe^2+^ complex. It has been known that iron was the determining factor for the initiation of lipid peroxidation [14]. Gawlik-Dziki *et al.* have observed that the 50% ethanolic extract from quinoa leaves prevented lipid oxidation which might be due to its ferrous ion chelating activity. Besides, the ethanolic extract of quinoa leaves showed significant inhibition of proliferation in prostate cancer cells (MAT-LyLu and AT-2) [15]. The quinoa leaf extracts used in our study acted as effective ferrous ion chelator which may consequently reduce the oxidative stress. Interestingly, the WQL showed a higher Fe^2+^ chelating potential in spite of its low DPPH activity. This observation suggests that the use of different methods is necessary to measure the antioxidant potentials of plant extracts.

Under normal physiological conditions, NO acts as a necessary component in the regulation of various physiological functions such as blood pressure, immune response, and neural communication [16]. However, overproduction of NO can induce tissue damage and is associated with inflammatory diseases including atherosclerosis and hypertension [17, 18]. Therefore, researchers have paid more attention to discovering natural antioxidants that may act as potent inhibitors of NO production in relation to the treatment of chronic inflammatory diseases [17]. Bacterial LPS are known to be activators of murine macrophage RAW 264.7 cells, which are a suitable model for evaluating the potential inhibition of NO production. In this study, the 50% EQL and 95% EQL were found to evidence an inhibitory activity against NO production in LPS-stimulated RAW 264.7 cells. These data are related to the higher level of total phenolic content in the ethanolic extracts when compared to the water extract. The phenolic compounds have long been considered to have the potential to inhibit NO and peroxynitrite production [19], indicating that the presence of antioxidant molecules in all the crude quinoa leaf extracts is responsible for their inhibitory effect. Previous epidemiological studies have indicated an inverse relationship between the consumption of antioxidant-rich food and the reduction of risk factors of some human diseases [20]. In addition, the secondary metabolite of plants has also been reported to act as excellent anti-inflammatory agent in oxidative stress and inflammation [21].

## 5. Conclusion

The results of the present study indicated that the 95% EQL contained the highest amount of total phenolic content and provided the greatest DPPH scavenging activity among all extracts, as well as inhibited NO production in LPS-induced RAW 264.7 cells. Our results suggest that quinoa leaves are a valuable bioactive natural product for dietary supplementation. Further studies *in vivo* are required to confirm these findings.

## Conflicts of interest

The authors declare no conflicts of interest.

## References

[1] Cai, Y, Luo Q, Sun M, Corke L. Antioxidant activity and phenolic compounds of 112 traditional Chinese medicinal plants associated with anticancer. Life Sci. 2004; 74: 2157-84. 14969719 10.1016/j.lfs.2003.09.047 PMC7126989

[2] Toyokuni, S, Tanaka T, Kawaguchi W, Fang NR, Ozeki M, Akatsuka S, et al Effects of the phenolic contents of Mauritian endemic plant extracts on promoter activities of antioxidant enzymes. Free Radic Res. 2003; 37: 1215-24. 14703734 10.1080/10715760310001598150

[3] Liu RH. Potential synergy of phytochemicals in cancer prevention: mechanism of action. J Nutr. 2004; 134: 3479S-85S. 15570057 10.1093/jn/134.12.3479S

[4] Kokanova-Nedialkova Z, Nedialkov P, Nikolov S. The genus che-nopodium: Phytochemistry, ethnopharmacology and pharmacology. Pharmacogn Rev. 2009; 3: 280-306.

[5] Bhargava, A, Shukla S, Ohri O. Chenopodium quinoa-An Indian perspective. Industrial Crops and Products. 2006; 23: 73-87.

[6] Gordillo-Bastidas E, Diaz-Rizzolo DA, Roura E, Massanes T, Gomis R. Quinoa (Chenopodium quinoa Willd.), from Nutritional Value to Potential Health Benefits: An Integrative Review. J Nutr Food Sci. 2016; 6: 497.

[7] Taga MS, Miller EE, Pratt DE. Chia seeds as a source of natural lipid antioxidants. J the Am Oil Chem Soc. 1984; 61: 928-31.

[8] Shimada, K, Fujikawa K, Yahara K, Nakamura T. Antioxidative properties of xanthan on the autoxidation of soybean oil in cyclodex-trin emulsion. J Agric Food Chem. 1992; 40: 945-48.

[9] Dinis TC, Maderia VM, Almeida LM. Action of phenolic derivatives (acetaminophen, salicylate, and 5-aminosalicylate) as inhibitors of membrane lipid peroxidation and as peroxyl radical scavengers. Arch Biochem Biophys. 1994; 315: 161-9. 7979394 10.1006/abbi.1994.1485

[10] Schafer FQ, Qian SY, Buettner GR. Iron and free radical oxidations in cell membranes. Cell Mol Biol. (Noisy-le-grand) 2000; 46: 657-62. 10872752 PMC3800086

[11] Wong CC, Li HB, Cheng KW, Chen F. A systematic survey of antioxidant activity of 30 Chinese medicinal plants using the ferric reducing antioxidant power assay. Food Chem. 2006; 97: 705-11.

[12] Ajayi AM, Tanayen JK, Magomere A, Ezeonwumelu JOC. An-tinociceptive and anti-inflammatory effects of aqueous extract of Chenopodium opulifolium (Italic) schrad leaves. J Intercult Ethnop-harmacol. 2017; 6: 14-21. 10.5455/jice.20161229055924 PMC5289083 28163955

[13] Fresco, P, Borges F, Diniz C, Marques MP. New insights on the an-ticancer properties of dietary polyphenols. Med Res Rev. 2006; 26: 747-66. 16710860 10.1002/med.20060

[14] Braughler JM, Duncan LA, Chase RL. The involvement of iron in lipid peroxidation. J Biol Chem. 1986; 261: 1028-29. 3015924

[15] Gawlik-Dziki U, Swieca M, Sulkowski M, Dziki D, Baraniak B, Czyz J. Antioxidant and anticancer activities of Chenopodium qui-noa leaves extracts - in vitro study. Food Chem Toxicol. 2013; 57: 154-60. 23537598 10.1016/j.fct.2013.03.023

[16] Moncada, S, Palmer RM, Higgs EA. Nitric oxide: physiology, pathophysiology, and pharmacology. Pharmacol Rev. 1991; 43: 109-42. 1852778

[17] Pacher, P, Beckman JS, Liaudet L. Nitric oxide and peroxynitrite in health and disease. Physiol Rev. 2007; 87: 315-424. 17237348 10.1152/physrev.00029.2006 PMC2248324

[18] Taira, J, Nanbu H, Ueda K. Nitric oxide-scavenging compounds in Agrimonia pilosa Ledeb on LPS-induced RAW264.7 macrophages. Food Chem. 2009; 115: 1221-27.

[19] Conforti, F, Menichini F. Phenolic compounds from plants as nitric oxide production inhibitors. Curr Med Chem. 2011; 18: 1137-45. 21291370 10.2174/092986711795029690

[20] Udenigwe CC, Lu YL, Han CH, Hou WC, Aluko RE. Flaxseed protein-derived peptide fractions: Antioxidant properties and inhibition of lipopolysaccharide-induced nitric oxide production in murine macrophages. Food Chemistry. 2009; 116: 277-84.

[21] Sheu, F, Lai HH, Yen GC. Suppression effect of soy isoflavones on nitric oxide production in RAW 264.7 macrophages. J Agric Food Chem. 2001; 49: 1767-72. 11308324 10.1021/jf001198+

